# Die Aufmerksamkeitsdefizit‑/Hyperaktivitätsstörung (ADHS) bei Erwachsenen in den klinischen Beschreibungen und klassifikatorischen Reflexionen von Gustav Specht (1905) und Hermann Paul Nitsche (1910)

**DOI:** 10.1007/s00115-021-01233-7

**Published:** 2021-11-24

**Authors:** Holger Steinberg, Maria Strauß

**Affiliations:** 1 Forschungsstelle für die Geschichte der Psychiatrie, Klinik und Poliklinik für Psychiatrie und Psychotherapie, Medizinische Fakultät, Universität Leipzig, Semmelweisstr. 10, 04103 Leipzig, Deutschland; 2 ADHS-Sprechstunde, Klinik und Poliklinik für Psychiatrie und Psychotherapie, Universitätsklinikum Leipzig AöR, Leipzig, Deutschland

**Keywords:** Aufmerksamkeitsdefizit‑/Hyperaktivitätsstörung (ADHS), Erwachsene, Geschichte der Psychiatrie, Klassifikation, Krankheitsentität, Adult attention-deficit/hyperactivity disorder (ADHD), Adult, History of psychiatry, Classification, Psychiatric entity

## Abstract

Es verstetigt sich die Ansicht, dass die adulte Form der Aufmerksamkeitsdefizit‑/Hyperaktivitätsstörung (ADHS) kein Konstrukt der modernen Psychiatrie ist. Die Geschichte der Psychiatrie kann hier einen aufklärenden Beitrag leisten. Diesem Anspruch und konkret Literaturverweisen Emil Kraepelins (1856–1926) folgend, wird hier jeweils eine Studie von Gustav Specht (1860–1940) und dem späteren NS-Psychiater Hermann Paul Nitsche (1876–1948) aus den Jahren 1905 bzw. 1910 zum Themenfeld chronische Manie inhaltlich analysiert. Wir gelangen zu dem Schluss, dass beide Autoren in mehreren Kasuistiken Kranke schildern und in ihre klinischen Erörterungen Aspekte einfließen lassen, die die heute so definierten Kernsymptome der adulten ADHS berühren oder in denen sich aktuell diskutierte Forschungsfragen wiederfinden. Beide Autoren drücken ihre Unzufriedenheit mit der klassifikatorischen Situation dieser Kranken in ihrer Zeit aus. Specht postuliert die „chronische Manie“, unter der er die adulten ADHS-Patienten einfasst, sogar als eine „völlig selbständige Geisteskrankheit“, die von den Psychiatern aber kaum als vollwertige Krankheitsform anerkannt, sondern eher als Verlegenheitsdiagnose verwendet werde. Nitsche sieht die von ihm sog. „chronisch-manischen Zustände“ zwar als „klinische Eigenart“, rechnet sie aber der großen Gruppe des „manisch-depressiven Irreseins“ zu. In Zukunft erst werde diese feiner aufzuteilen sein.

## Abstract (en)

The notion that the adult form of attention deficit hyperactivity disorder (ADHD) is not a construct of modern psychiatry is increasingly prevailing. Looking into the history of psychiatry can make an enlightening contribution here. Guided by this aim and specifically following literature referred to by Emil Kraepelin (1856–1926), we analyzed the content of one study each by Gustav Specht (1860–1940) and the later Nazi psychiatrist Hermann Paul Nitsche (1876–1948) from 1905 and 1910, respectively, on the topic of chronic mania. Our investigation concluded that in their case studies both authors described people who would today be diagnosed as suffering from adult ADHD as the clinical descriptions reveal core symptoms of this entity as defined by modern classifications. They also mentioned currently discussed research questions. Both authors expressed their dissatisfaction with the classificatory situation of these patients at the time. Specht even postulated a “completely independent mental illness” that he called “chronic mania”, under which he classified all the patients suffering from today’s adult ADHD. He also pointed out that this diagnosis was not widely recognized at the time by psychiatrists as a full-fledged form of illness but used more as a diagnosis to avoid the embarrassment of not having one. Nitsche saw the “chronic manic states” as he called them as a “clinical peculiarity” but assigned them to the large group of “manic depressive insanity”, which could only be more finely differentiated in the future.

Die Erkenntnis, dass die ADHS bei Erwachsenen keine neue Erscheinung der letzten Jahrzehnte darstellt, obgleich sie erst 1980 erstmals als diagnostische Kategorie im DSM-III (Diagnostic and Statistical Manual of Mental Disorders-III) als „ADD Residual Type“ aufgenommen wurde, verstetigt sich. Die geschichtswissenschaftliche Aufarbeitung des theoretischen Denkens und praktischen Tuns vorheriger Generationen in den psychiatrischen Einrichtungen kann wesentlich zu diesem Wissen beitragen. Diesem Anspruch folgend werden zwei Studien der prominenten Psychiater Gustav Specht (1860–1940) und Hermann Paul Nitsche (1876–1948) aus den Jahren nach 1900 vorgestellt und inhaltlich mit gegenwärtig verbreiteten Diagnostikmanualen hinsichtlich der adulten ADHS kontrastiert. Nitsche, einer der theoretischen Denker, aber auch direkt handelnden Akteure der NS-„Euthanasie“ und des hunderttausendfachen Mordens an Patienten, war vordem ein progressiver Anstaltspsychiater und hinterließ ein umfangreiches, das unmittelbar Wissenschaftlich-Klinische des Faches betreffende Schrifttum. Dieses zu sichten, zu analysieren und einzuschätzen bleibt trotz der enormen Schuld, die Nitsche auf sich geladen hat, auch Aufgabe der psychiatriehistorischen Forschung.

## Erste historische Spuren

Einen gelungenen Gesamtüberblick zur Geschichte der ADHS bieten z. B. Martinez-Badía und Martinez-Raga [19]. Im Gegensatz zu vielen vorliegenden historischen Untersuchungen zur kindlichen Form der ADHS muss die Geschichte der ADHS bei Erwachsenen jedoch nach wie vor als nahezu unaufgearbeitet gelten. Eine erste historische Kurzübersicht [5] folgt der Entdeckung [4], dass der Fuldaer Arzt Melchior Adam Weikard (1742–1803) 1775 in einer seiner anonym unter dem Titel „Der philosophische Arzt“ [2] erschienenen Schriften über Symptome der ADHS auch bei Erwachsenen berichtete. Dies gilt bislang als Erstbeschreibung. Nachdem in der Kurzübersicht bereits für das Jahr 1798 [10] der Schotte Alexander Crichton (1763–1856) folgt, wird noch der Londoner Kinderarzt George Still (1868–1941) gewürdigt. Allerdings fällt sein Beitrag schon in den Beginn des 20. Jahrhunderts [27].

Zuletzt wurde auf die psychiatriehistorisch außerordentlich bedeutsame 8. Auflage des Psychiatrielehrbuches von Emil Kraepelin (1856–1926) aus den Jahren 1909 bis 1915 hingewiesen [17, 26]. Diese Arbeit gelangte zu der Auffassung, dass Kraepelin in seiner klinischen Praxis erwachsene Patienten gesehen habe, die heute die Diagnose ADHS erhalten würden. So ließe sich in zwei in seinem Lehrwerk aufgeführten Entitäten das heute definierte Krankheitsbild ADHS bei Erwachsenen wiederfinden. Einerseits in einem „Grundzustand“ des „manisch-depressiven Irreseins“, den er als „manische Veranlagung“ oder auch als „konstitutionelle Erregung“ bezeichnete, und andererseits in der Gruppe der „Haltlosen“, die er als eine Form der psychopathischen Persönlichkeiten klassifizierte. Graduell leichtere Formen mit vordergründig ADHS-assoziierten Stimmungsschwankungen habe er der „manischen Veranlagung“ zugeordnet und schwerere Formen, welche durch Komorbiditäten wie ausgeprägte Persönlichkeitsstörungen und Suchterkrankungen gekennzeichnet seien, habe er den „Haltlosen“ zugewiesen [26]. Eine Zusammenführung all dieser Patienten in einer eigenständigen nosologisch postulierten Entität nahm der Lehrbuchautor allerdings nicht vor. Es muss jedoch, um unserem Anliegen, Hinweise auf Spuren erwachsener ADHS-Patienten in vergangenen Zeiten zu finden und kritisch zu prüfen, zu entsprechen, höchst aufmerken lassen, dass Kraepelin bei der Beschreibung seiner beiden Krankheitsformen nicht nur auf die eigene Erfahrung, sondern auch auf Berichte nervenärztlicher Kollegen verweist. So führt er in die Darstellung der „konstitutionellen Erregung“ ein, dass er befindet, diese sei „in neuerer Zeit besonders von Specht und Nitsche genauer geschildert worden“ [17, Bd. 3, S. 1312].

## Gustav Specht und die „Chronische Manie und Paranoia“ von 1905

Bei Gustav Specht handelt es sich um Kraepelins Erlanger psychiatrischen Lehrstuhlkollegen und ersten Direktor der dortigen, 1903 aus einem Teil der Heil- und Pflegeanstalt heraus gegründeten Universitäts-Nervenklinik. 1896 war der gebürtige Schweinfurter noch als Oberarzt der mittelfränkischen Kreisirrenanstalt, wo er 11 Jahre zuvor als Arzt eingetreten war, zum außerordentlichen Professor berufen worden. Ordinariat und Direktorat sollte er bis 1934 innehaben. Den Höhepunkt seiner akademischen Karriere hatte er zuvor 1913/1914 als Prorektor der Friedrich-Alexander-Universität Erlangen erlebt.

Specht wird in einem sehr persönlichen Nachruf von seinem Schüler Karl Kleist (1879–1960) 1941 als „ein kundiger und gütiger Seelenarzt, ein gedankenreicher Forscher, ein vortrefflicher Lehrer“ gewürdigt [15, S. 238]. Zudem kann er als ein bedeutender, nachwirkender Kliniker seiner Zeit bis heute gelten, wirkte er doch sogar schulbildend. Die heute sog. klassische „Erlanger Schule“ wurde von ihm begründet [9] und über seine Schüler und Mitarbeiter fortgeführt, von denen vor allem Kleist, Gottfried Ewald (1888–1963) und Karl Leonhard (1904–1988) sowie die beiden Anstaltsdirektoren Josef Klüber (1873–1936) und Valentin Faltlhauser (1876–1961) größere Bekanntheit erlangten. Letzterer war allerdings später T4-Gutachter und an Euthanasieverbrechen beteiligt. Die „Erlanger Schule“ wurde demzufolge als entscheidendes Bindeglied in der Entwicklung der Wernicke-Kleist-Leonhard-Schule dargestellt [9]. Spechts „offenkundige Bevorzugung“ [15, S. 235] in seiner wissenschaftlichen Arbeit galt den affektiven und paranoischen Erkrankungen, die sich nach seiner Lesart auch oft überlappen würden. Vielleicht besteht Spechts bedeutendster Beitrag zur Konzeptgeschichte des Fachs in seiner Abhandlung über den Angstaffekt im „manisch-depressiven Irresein“. Dafür hatte er Carl Wernickes (1848–1905) Beschreibung der „Angstpsychose“ aufgegriffen und regte Karl Leonhard zu dessen späterer Konzeption der „Angst-Glücks-Psychose“ an [9]. Kleist sieht indes in der „Vertiefung und Verfeinerung des Krankheitsbildes der chronischen Manie an sich“ Spechts wertvollsten Beitrag zum Fach. Er habe die chronische Manie als einen konstitutionellen psychopathischen, sich verschlimmernden Zustand erkannt und von phasischen Verlaufsformen abgegrenzt [15, S. 231–232].

Zwischen Specht und Kraepelin lassen sich immer wieder Berührungspunkte finden. So integrierte Specht z. B. Kraepelins Querulantenwahn in sein eigenes Konzept der „chronischen Manie“ [25]. Diejenige Arbeit Spechts aber, auf die Kraepelin in der 8. Auflage seines Lehrbuchs hinweist und die dessen Konzept der „chronischen Manie“ vorstellt, ist sein im Jahr 1905 erschienener Aufsatz über „Chronische Manie und Paranoia“ im *Centralblatt für Nervenheilkunde und Psychiatrie*. Specht beginnt seine Ausführungen dort mit dem Hinweis, dass die „chronische Manie“ als „verschwommene Verlegenheitsdiagnose“ [24, S. 590] missbraucht werde und dass sie „in der Bedeutung einer vollwertigen und durchaus selbstständigen Krankheitsform noch kein bleibendes Heimatsrecht zu erwerben vermocht“ habe [24, S. 591]. Weiterhin betont er, dass die echte „chronische Manie“ vom sekundären manischen Dauerzustand unbedingt zu trennen sei und dass es sich bei ihr um eine „völlig selbstständige Geisteskrankheit“ handele, welche „nicht zu den Raritäten gehört“ [24, S. 591]. Die „chronische Manie“ sei seines Wissens bislang nur von seinen Kollegen Wernicke, Julius Ludwig August Koch (1841–1908), Ernst Siefert (1874–1940) und Kraepelin als solche gewürdigt worden. Der symptomatologische Kern der Krankheit werde seiner Meinung nach durch den „manischen Erscheinungskomplex“ gebildet, allerdings auf der niedrigen Intensitätsstufe der Hypomanie, aber mit möglichem temporärem Exazerbationspotenzial, bis hin zu den Erscheinungsbildern einer „furibunden Tobsucht“ oder einer „deliranten Verworrenheit“. Neben den Kernsymptomen sieht er aber eine Reihe sekundärer Erscheinungen, die er als natürliche Folge der Chronizität interpretiert. Der Beginn des psychopathologischen Zustands der „chronischen Manie“ falle gemäß seiner Beschreibung mit der „Ausreifung der Persönlichkeit“ zusammen, was einem ADHS-typischen Beginn in der Kindheit nahekommt. Weiterhin beschreibt er die Betroffenen als „Sprösslinge psychopathisch durchseuchter Geschlechter“, was für eine Erblichkeit der Störung spricht, und erwähnt Berufe wie „Marktschreier“ oder „Spassmacher“, denen Betroffene oft nachzugehen scheinen. Auch beschreibt er sehr treffend, dass bei dieser Störung unterschiedliche Ausprägungen zwischen Gesund und Krank existierten und manche sogar als Grenzfälle einzuordnen seien, welche „auch dem Fachmann zuweilen diagnostisches Kopfzerbrechen verursachen“. Er sieht eindeutig ein diagnostisches Problem für diese für ihn klar greifbare Krankheitsentität und spricht sogar von Missdeutung der „ausgeprägten Form der chronischen Manie … durch Jahre und Jahrzehnte“. In diesem Zusammenhang stellt er sich die Frage, „wo steckte die chronische Manie bisher und wo steckt sie bei jenen Psychiatern, die sie noch nicht kennen oder überhaupt nicht anerkennen“. Er wähnt sie als „chronische Paranoia“, „chronischen Alkoholismus“ oder „epileptisches Irresein“ fehldiagnostiziert. Insbesondere die Abgrenzung zur „chronischen Paranoia“ erscheint ihm sehr wichtig, zumindest widmet er einer dahingehenden Argumentation einen längeren Abschnitt. Schließlich resümiert er, bei der chronischen Manie handele es sich um „eine nicht selten vorkommende, durchaus selbständige und“ – im Einklang mit Kraepelin – „den konstitutionellen Geistesstörungen zugehörige Krankheitsform, die nicht mit gewissen Endstadien anderer Psychosen … oder unklaren Aufregungszuständen zusammengeworfen werden darf“, und die als Entität von der Fachwelt eine eindeutigere Würdigung verdiene [24].

## Hermann Paul Nitsche und die „Chronisch-manischen Zustände“ von 1910

Der Aufsatz über die „chronisch-manischen Zustände“ von Nitsche, auf den Kraepelin in seinem Lehrbuch hingewiesen hatte, umfasst nahezu 100 Seiten und war 1910 in dem verbreitetsten deutschsprachigen, klinisch-anwendungspraktisch orientierten psychiatrischen Fachblatt, der *Allgemeinen Zeitschrift für Psychiatrie und psychisch-gerichtliche Medizin* erschienen. Kraepelin kannte Nitsche persönlich gut und schätzte ihn, immerhin hatte er ihm, dem Assistenzarzt seiner Heidelberger Psychiatrischen Universitätsklinik, das Angebot unterbreitet, mit ihm 1904 nach München an die dortige Psychiatrische Universitätsklinik in der Nußbaumstraße zu kommen und ihn dort zum Ersten Assistenten befördert. 1908 war der gebürtige Colditzer dann jedoch zurück in seine sächsische Heimat gekehrt. Zum Zeitpunkt der Veröffentlichung des Aufsatzes war er Oberarzt der Heil- und Pflegeanstalt Dresden-Löbtau, im Laufe der folgenden Jahre sollte er mehrfach wechselnd die Stellen des Direktors der Heil- und Pflegeanstalten Pirna-Sonnenstein und Leipzig-Dösen besetzen. War in Beurteilungen über den jungen Arzt „sein Talent sowie die Herzlichkeit und Geduld im Umgang mit seinen Patienten“ [8] oder die auf Zwangsmittel und „jede schematisierende Behandlung“ verzichtende, „sich speziell mit besonders schwer zu behandelnden, namentlich erregten Kranken alle nur erdenkliche Mühe“ gebende „vorzügliche Tätigkeit“ gelobt worden, sodass er „bei den Kranken außerordentlich beliebt“ war, sie ihm mit „Liebe und Dankbarkeit“ anhingen [1, S. 55], ließen gleichzeitig seine reformpsychiatrischen Konzepte aufmerken. So praktizierte er in seinen Anstalten anstatt stationärer die Familienpflege für gebesserte oder die aktive Beschäftigungstherapie nach Simon für chronische Patienten, auch sein frühzeitiges Engagement für eine Heilung von progressiver Paralyse durch die neue Malariatherapie könnte als Hinweis auf sein ärztliches Ethos gelesen werden. Gleichzeitig stand er jedoch der Rassenhygiene sehr nahe. Die freiwillige Sterilisation bei eindeutig gesetzlich geregelter ärztlicher Gutachtertätigkeit führte er in den 1920er-Jahren als sozial und allgemein prophylaktisch als „dringend wünschenswert“ bei einer „sicher diagnostizierten und sicher vererbbaren geistigen Störung“ an [21]. Im Kontext wird klar, dass er aber weitergehende rechtliche Grundlagen auch für Zwangssterilisationen und Eheverbote „Geisteskranker“ einforderte. Nach Hitlers Machtergreifung enthemmten sich Nitsches Äußerungen, er begrüßte das „Gesetz zur Verhütung erbkranken Nachwuchses und die Vernichtung lebensunwerten Lebens“ und sollte auch selbst einer der ärztlichen Haupttäter bei den Verbrechen an hilflosen und leidenden Menschen werden. Er führte 1936 in Pirna-Sonnenstein die Hungerkost für Patienten ein, die nach seinen Vorstellungen als „Ballastexistenzen“ anzusehen waren. In Vorbereitung der Aktion-T4, der systematischen Erfassung und Vergasung kranker und behinderter Menschen zwischen 1940 und 1941 in Tötungsanstalten, entwickelte und erprobte Nitsche an über 100 Patienten das Barbiturat Luminal als Tötungsmittel. Zunächst als T4-Gutachter engagiert, rückte er ab Mai 1940 direkt in die Spitze der T4-Organisation vor und fungierte als Obergutachter und ab Dezember 1941 als medizinischer Leiter. Auch an der sowohl sich anschließenden „wilden Euthanasie“, also der zumeist medikamentösen Tötung Kranker in den Anstalten vor Ort, als auch an der Aktion 14f13, der Vergasung von KZ-Häftlingen, war Nitsche beteiligt. Er trug somit Verantwortung für die Ermordung von weit mehr als 100.000 Menschen und wurde folgerichtig im sog. Dresdner „Euthanasie“-Prozess 1947 zum Tode verurteilt. Am 25.03.1948 wurde durch das Fallbeil das Strafmaß vollstreckt [1, 8, 18, 23]. Als widersprüchlich muss die Entwicklung Nitsches vom humanen Reformpsychiater zum Arzt und Bürokrat, der tötete, erscheinen. Jedoch muss man sehen, dass Nitsche noch unter den gesetzlichen und moralischen Beschränkungen der Weimarer Republik aktiv Anstöße gegeben hatte, die Anstalten effektiver und ökonomischer zu machen. Diese Anstöße können auch als Kontinuum von Gedanken gelesen werden, die im rechtsfreien Raum nach 1933 in teilweise extremste Radikalisierungen der schon lange diskutierten sozialdarwinistischen Lehren mündeten. Damit einhergehend setzte sich seine auf das individuelle Patientenschicksal gerichtete Heils- und Humanitätsmoral für ihn fort als Eugenik, wurde aus seiner therapeutischen eine prophylaktische, das gesamte Kollektiv der „Deutscharier“ schützende Bevölkerungs- und Gesundheitspolitik. Er selbst sah sich konsequent fortschreiten auf dem Weg zur Heilung der „Geisteskrankheiten“. Zwangsläufig verlief eine solche radikale Entwicklung freilich nicht, andere Reformärzte wurden nicht zu Massenmördern.

Nitsche beginnt seinen hier vorzustellenden Beitrag „Über chronisch-manische Zustände“ mit der Feststellung, dass man auf eben diese „erst vor kurzem aufmerksam geworden sei“. Er kündigt an, die „bislang spärliche Kasuistik“ zu dieser „klinischen Eigenart“ durch Mitteilung „von im Laufe der Zeit von mir gesammelten Krankengeschichten“ bereichern zu wollen. Obgleich er die Stellung seiner klinischen Fälle dem „manisch-depressiven Irresein“ zurechnen würde, wage er gleichzeitig die Annahme, dass es sich bei dieser Einteilung „nur um eine Durchgangsphase … des Zusammenwerfens“ handele, welcher eine „Phase des Sichtens und Abteilens“ in Zukunft folgen werde [20, S. 37]. Darauf folgend gibt er einen Überblick über den „jetzigen Stand der Lehre von der chronischen Manie“, welcher die Meinung von „neueren Forschern“ wie Theodor Ziehen (1862–1950), Adolf Schott (vor 1874–nach 1934), Wernicke und Specht widerspiegelt, und den er dann mittels der Betrachtung der „hierher gehörigen Krankheitsfälle“ substantiiert [20].

Anhand der Symptome ordnet er die Fälle in vier Gruppen ein. Der ersten Gruppe ordnet er zwei Fälle von Siefert zu, deren Typus er als „originäre Hypomanie“ bezeichnet. Nitsche stellt hierbei fest, „die Symptomatik unterscheide sich in nichts von den hypomanischen Phasen des manisch-depressiven Irreseins, jedoch bestehe dieser Zustand von Jugend auf … ohne jedoch, dass das ununterbrochene Bestehen des hypomanischen Zustandes exakt bewiesen sei“ [20, S. 117]. Die zweite Gruppe „umfasse Kranke, die von Natur aus sanguinische, selbstbewusste, unbeständige, unternehmungssüchtige Menschen seien“. Da sich der Zustand dieser Patienten „gegen das dritte oder vierte Jahrzehnt hin zu einer leichten Erregung steigere und um das 50. Lebensjahr eine deutliche hypomanische Psychose einsetze“, welche sich in einem Fall sogar bis zu einer schweren Manie gesteigert habe, bezeichnet Nitsche diese Fälle als „progressive manische Konstitution“. Auch bei der dritten Gruppe träten die Symptome „im reiferen Alter, nämlich im 35. und 42. Lebensjahr“ auf. Für diese Patienten sei „ein hypomanischer Zustand von abnorm langer Dauer als Phase eines zirkulären Irreseins“ kennzeichnend [20].

In der vierten Gruppe finden sich die Fälle mit „manischer Erregung“, welche in ihrer Ausprägung „einem leichtesten, nicht psychotisch wirkenden Grade“ entsprechen. Diese Kranken würden meistens eine „leichteste motorische Erregung, einen minderwertigen Betätigungsdrang, teilweise auch soziale Unstätheit, Reizbarkeit, Neigung zum Querulieren“ zeigen. Diese vierte Patientengruppe bereitete bezüglich ihrer diagnostischen Zugehörigkeit Nitzsche offensichtlich am meisten Kopfzerbrechen, was seine Formulierung „es fragt sich nun, wie die symptomatisch leichtesten Formen (konstitutionelle Erregung Kraepelins, manische Verstimmung Jungs^1^) aufzufassen sind“, eindeutig wiedergibt. Er resümiert, dass man wohl eine „einheitliche, allen Fällen gerecht werdende Schilderung vom Symptombilde der chronischen Manie“ anhand der beschriebenen Krankheitsfälle nicht für möglich halte [20, S. 124]. Er sehe aber einige gemeinsame Merkmale, welche häufiger bei den chronisch verlaufenden Formen manischer Zustände aufzutreten scheinen. Hierzu zähle er Ablenkbarkeit, Weitschweifigkeit und Erinnerungsfälschungen. Außerdem vermutete er bei dieser Patientengruppe eher „vorübergehende reaktive Schwankungen bei sehr labiler Affektlage“ als „streng endogene psychotische Verstimmungen“. Zusätzlich sehe er bei der Großzahl der Krankheitsfälle eine „erbliche Belastung“ [20, S. 125–126].

Insgesamt glaube er nicht, dass man verlässliche Angaben zur Häufigkeit „chronisch manischer Zustände“ aufgrund der ihm vorliegenden Erkenntnisse machen könne, da die leichten Fälle, welche er als „Fälle von konstitutioneller Erregung“ einordnet, selten in die Anstalten kämen. Gleichzeitig vermute er, dass genau diese Fälle recht häufig seien. Prognostisch schienen diese leichteren Formen die Neigung zu haben, „als dauernde Eigentümlichkeit zu bestehen und es gehört eine Heilung … wohl zu den Seltenheiten“ [20, S. 127]. Sehr wichtig erscheine ihm bei diesen leichteren Fällen die differenzialdiagnostische Abgrenzung aufgrund der „vielseitigen Beziehungen der konstitutionellen Erregung zu dem Gebiete der Grenzzustände“ [20, S. 129].

Nitsche versucht, seine Beobachtungen mittels einiger Krankengeschichten zu verdeutlichen. Exemplarisch sei hier der Fall des Studenten Karl Dörfler erwähnt. In der Schule habe sich dieser zerstreut gezeigt und es seien „mangelhafter Fleiß und die Ungleichmäßigkeit seiner Leistungen“ beklagt worden. Außerdem sei er „unordentlich“ gewesen. Als weitere Kennzeichen führt Nitsche das Unvermögen, Tätigkeiten „zu Ende zu führen“ und „die Rastlosigkeit seiner Gedanken“ an. Außerdem sei Dörfler durch eine „dauernde leichte motorische Erregung“ aufgefallen und habe der Patient bei seinen Bewegungen eine große Hast gezeigt, habe auch überstürzt gesprochen. Zusätzlich nahm der Arzt eine heftige innere Spannung wahr. Auffallend sei schließlich auch der bisherige Lebenslauf des Studenten gewesen. In der Schule seien Konflikte mit Mitschülern und bei der Armee mit den Vorgesetzen entstanden. Er habe sogar Fahnenflucht begangen. Ausbildungen habe er nach wenigen Monaten wieder abgebrochen und soll sich für mehrere Studienfächer gleichzeitig eingeschrieben haben. Auch habe er mehrere Gerichtsverhandlungen wegen Schulden und Streitigkeiten führen müssen [20, S. 21, 82–86].

## Diskussion

Trotz der hohen Prävalenz von bis zu 2,8 % [13] ist die ADHS im Erwachsenenalter nach wie vor außerhalb spezialisierter Ambulanzen wenig bekannt und spielt im allgemein-psychiatrischen Kontext für erwachsene Patienten noch eine untergeordnete Rolle. Obwohl ein ADHS-spezifischer psychopathologischer Befund vorliegt, der eine Abgrenzung zu anderen psychischen Störungsbildern ermöglicht, wird die ADHS häufig als „Modeerscheinung“ und „neu eingeführtes Krankheitsbild“ eingestuft. Zugegebenermaßen gibt es einige Besonderheiten bei der Untersuchung erwachsener ADHS-Betroffener, welche die korrekte Diagnosestellung erschweren und eine gewisse Erfahrung mit diesem Störungsbild voraussetzen. Hierzu zählt die Überlappung ADHS-typischer Symptome wie Konzentrationsstörungen und Impulsivität mit denen anderer psychischer Störungen (z. B. aus dem affektiven Formenkreis), aber auch die hohe Anzahl an psychiatrischen Komorbiditäten, welche differenzialdiagnostische Überlegungen nötig macht [22]. Dies scheint auch Specht aufgefallen zu sein, der die ADHS-Betroffenen im Spektrum der „chronischen Manie“ einordnet und anmerkt, „trotz der überraschend spärlichen Bearbeitung, die darnach die chronische Manie bisher in der Literatur gefunden [hat – d. A.], ist sie … keine seltene Erkrankung und jeder beschäftigte Irrenarzt wird sicher alljährlich ein paar neue hierher gehörige Fälle zu Gesicht bekommen, wenn er sie nur erst einmal von anderen Psychosen zu differenzieren gelernt hat“ [24, S. 592]. Dies impliziert, dass ihm durchaus klargeworden ist, dass eine Reihe Betroffener mit dieser speziellen Symptomatik nicht als solche und nicht als zu einer definierbaren Gruppe gehörig erkannt wird, weil die „Irrenärzte“ nicht über eine ausreichende Kenntnis des psychopathologischen Befunds verfügen. Dies entspricht auch der aktuellen Problematik von ADHS-Betroffenen, welche häufig unterdiagnostiziert und somit unbehandelt bleiben [3, 14]. Als besonders bemerkenswert und wegweisend für unsere Annahme, dass Gustav Specht in seinen Beschreibungen ADHS-Patienten meinte, kann folgende seiner Aussagen gelten: „Die vielberufene Grenzlinie zwischen Gesund und Krank ist gerade hier bis zur Unkenntlichkeit verwischt und man muss, zumal in den milderen Fällen, seinem Gutachten eine ganz besondere Überzeugungskraft zu verleihen suchen, um dem billigen Vorwurf, als könne sich die Psychiatrie in der Schaffung neuer Krankenspezialitäten nicht genug tun, nicht noch weitere Nahrung zu geben“ [24, S. 593]. Offensichtlich gab es auch zu seiner Zeit um 1900 in der Öffentlichkeit sowie auch unter der Ärzteschaft den Eifer, über die Neuschaffung von Diagnosen in der Psychiatrie zu sprechen, was genau einer häufigen medialen Diskussionslinie über ADHS im Erwachsenenalter unserer Zeit gleichkommt, welche postuliert, dass ADHS eine Modeerscheinung sei und keinen echten Krankheitswert besitze (Tab. 1).

**Table 1:** 

Autor	Aufmerksamkeitsstörung	Motorische Überaktivität	Impulsivität	Beginn in der Kindheit
Gustav Specht (1905)	„… durch die Ruhelosigkeit und Sprunghaftigkeit im Denken …“	„… in einer weniger intensiven Ausprägung der manischen Kardinalsymptome …“	„… expansive Stimmungsrichtung …“; „… aus der Leidenschaft geborenen Urteilsentgleisungen …“	„Die Entstehung des psychopathologischen Zustandes wird wohl immer mit der Ausreifung der Persönlichkeit zusammenfallen …“
Hermann Paul Nitsche (1910)	„… mangelhafter Fleiß und die Ungleichmäßigkeit seiner Leistungen …“; „… in seiner Rede schweifte er leicht ab …“; „Unvermögen, Tätigkeiten zu Ende zu führen“	„... dauernde leichte motorische Erregung …“; „… er gestikulierte beim reden lebhaft …rutschte auf dem Stuhle hin und her …“	„... sei reizbar und zornig …“; „… leichtsinnig… unbedenklich in der Wahl seines Umgangs …“	„In der Schule habe sich dieser zerstreut gezeigt …“

*ADHS* Aufmerksamketisdefizit‑/Hyperaktivitätsstörung

Auch Nitsche beschreibt in seinen Ausführungen „Über chronisch-manische Zustände“ eine Patientengruppe, welche sehr stark an adulte ADHS-Patienten erinnert. Er selbst ordnet diese Fälle diagnostisch zwar dem „manisch-depressiven Irresein“ zu, meldet aber gleichzeitig Zweifel an, ob dies langfristig die richtige Klassifizierung sei. Vor allem die von ihm als „leichtere Fälle“ bezeichneten Patienten weisen Symptome wie Weitschweifigkeit, Ablenkbarkeit und motorische Hyperaktivität auf, welche aus heutiger Sicht den ADHS-Kernsymptomen [11] zu entsprechen scheinen. Ein weiteres Argument dafür, dass Nitsche mit großer Wahrscheinlichkeit ADHS-Patienten meint, ist seine Beobachtung, dass sie zusätzlich unter einer vorübergehenden labilen Affektlage zu leiden scheinen, welche sehr treffend das Symptom der affektiven Dysregulation bei adulten ADHS-Patienten beschreibt. Nach aktueller Lehrmeinung leiden bis zu 70 % der Betroffenen unter dieser Symptomatik und es wird dringend empfohlen, bei der Diagnosestellung neben den Kernsymptomen Aufmerksamkeitsstörung, motorische Hyperaktivität und Impulsivität auch dieses Symptom zu berücksichtigen [6]. Des Weiteren fällt Nitsche eine „erbliche Belastung“ auf. Mittlerweile wissen wir, dass die ADHS familiär gehäuft auftritt und dass Verwandte 1. Grades ein 5‑ bis 10-fach erhöhtes Erkrankungsrisiko aufweisen [12]. Seine Beobachtungen und Überlegungen verdeutlicht Nitsche in mehreren kasuistisch dargestellten Krankheitsfällen. Vor allem in den Kasuistiken der von ihm postulierten vierten Patientengruppe werden nicht nur die typischen ADHS-Symptome, sondern auch die dadurch verursachten Funktionseinschränkungen in den unterschiedlichen Lebensbereichen wie Beruf und Ausbildung anschaulich dargestellt. Diese weiterreichende Problemsicht ist auch aus heutiger Sicht für die diagnostische Einschätzung von zentraler Bedeutung, da der Behandlungsbedarf nicht nur an das Vorhandensein einzelner Symptome, sondern anhand der verursachten Einschränkungen gemessen wird [28]. Ähnlich wie der Student aus Nitsches Falldarstellung erreichen auch heutzutage ADHS-Betroffene in der Summe niedrigere schulische und akademische Abschlüsse, haben Probleme im beruflichen Umfeld und in zwischenmenschlichen Beziehungen [7, 16]. Bemerkenswert erscheint dabei, dass damals wie heute die Symptomatik zu ähnlichen Problemkonstellationen für die Betroffenen geführt hat. Hieraus könnte geschlussfolgert werden, dass weniger die gesellschaftlichen und sozioökomischen Umstände die ADHS zu einer relevanten psychischen Störung machen, sondern dass wir es mit einer über die menschlichen Zeitalter konstant auftretenden Störung zu tun haben. Mögen die Kranken indes im vorindustriellen Zeitalter mit ihren Copingstrategien erfolgreicher und damit für ihre Umwelt unauffälliger geblieben sein. Insgesamt ergibt sich, dass Nitsches Aufsatz für die Rekonstruktion einer Ideengeschichte der adulten ADHS für die Jahre um 1900 ein wesentlicher Baustein sein könnte. Neben seinen bereits untersuchten Schriften über den Umbau des Anstaltswesens nach ausgesprochen reformpsychiatrischen Grundsätzen scheint es nun von daher angezeigt, seine im engeren Sinne wissenschaftlich-klinischen Publikationen in die Nitsche-, aber auch allgemein psychiatriehistorische Forschung einzubeziehen. Dies bedeutet in keinem Fall eine moralische Rehabilitation seiner Person oder eine Relativierung seiner Verbrechen.

Einhergehend mit dem Befund, dass Kraepelin in seinem Lehrbuch ganz offenbar erwachsene Patienten schildert, die an ADHS litten [26], und den hier beigebrachten Schilderungen von Specht und Nitsche, die ihrerseits wiederum auf andere zeitgenössische Kollegen verweisen, die über derartige Patienten berichten, verstetigt sich immanent die Auffassung, dass es sich bei der adulten Form der ADHS um ein konstant existentes Krankheitsphänomen über zumindest einen größeren Abschnitt in der neueren und neuesten Geschichte der Menschheit in hochzivilisierten Gesellschaften hinweg handelt. Weitere psychiatriehistorische Forschungen, die zunächst den Sekundärquellen folgen könnten, die die nunmehr analysierten Texte von Kraepelin, Specht und Nitsche angeben, scheinen dazu geeignet, die Korrektheit unserer Hypothese zu untersuchen. Für die Befunderhebung über die Epoche des wilhelminischen Deutschen Reichs hinaus, könnte aber als nächster Schritt die Analyse von Arbeiten von Nervenärzten zu Beginn der wissenschaftlichen Psychiatrie, also aus dem ersten Drittel des 19. Jahrhunderts aufschlussreich sein. Einerseits liegen von diesen Lehrwerke, Studien und Kasuistiken vor, die unserem Verständnis einer wissenschaftlichen Erkenntnisabsicht, Methodik und Darstellungsweise halbwegs folgen, und andererseits würden diese Quellen aus dem sog. vorindustriellen Zeitalter stammen, woraus Aufschlüsse hinsichtlich der Existenz und weiterfolgend des Wandels des zivilisatorisch-gesellschaftlichen Raums für dieses Krankheitsbild gezogen werden könnten.

## Footnotes

1 Nitsche rekurriert hier auf Carl Gustav Jungs (1875–1961) Aufsatz „Über manische Verstimmung“ (Allg Z Psychiatr 1902; 61:15–39).
